# SNCA 3′ UTR Genetic Variants in Patients with Parkinson’s Disease

**DOI:** 10.3390/biom11121799

**Published:** 2021-11-30

**Authors:** Antonela Blažeković, Kristina Gotovac Jerčić, Fran Borovečki

**Affiliations:** 1 Department for Functional Genomics, Center for Translational and Clinical Research, University of Zagreb School of Medicine, University Hospital Center Zagreb, 10000 Zagreb, Croatia; kristina.gotovac@mef.hr (K.G.J.); fbor@mef.hr (F.B.); 2 Department for Anatomy and Clinical Anatomy, University of Zagreb School of Medicine, 10000 Zagreb, Croatia; 3 Department of Neurology, University Hospital Center Zagreb, 10000 Zagreb, Croatia

**Keywords:** Parkinson’s disease, 3′ UTR, alpha-synuclein, PINK1, linkage disequilibrium, risk loci, next generation sequencing, single nucleotide polymorphism

## Abstract

The SNCA (Synuclein Alpha) gene represents a major risk gene for Parkinson’s disease (PD) and SNCA polymorphisms have been associated with the common sporadic form of PD. Numerous Genome-Wide Association Studies showed strong signals located in the SNCA 3′ UTR (untranslated region) region indicating that variants in 3′ UTRs of PD-associated genes could contribute to neurodegeneration and may regulate the risk for PD. Genetic variants in 3′ UTR can affect miRNA activity and consequently change the translation process. The aim of this study was to access the differences in 3′ UTR variants of SNCA genes in a cohort of PD patients and control subjects from Croatia. The cohort consisted of 52 PD patients and 23 healthy control subjects. Differences between 3′ UTR allele and genotype frequencies were accessed through next generation sequencing approach from whole blood samples. In our study, we identified four previously reported single nucleotide polymorphisms (SNPs) and one insertion in the 3′ UTR region of SNCA gene, namely rs1045722, rs3857053, rs577490090, rs356165, and rs777296100, and five variants not reported in the literature, namely rs35270750, rs529553259, rs377356638, rs571454522, and rs750347645. Our results indicate a significantly higher occurrence of the rs571454522 variant in the PD population. To the best of our knowledge, this variant has not been reported until now in the literature. We analyzed our results in the context of previous research, creating a brief overview of the importance of 3′ UTR variants of the SNCA gene. Further studies will be needed to gain a more profound insight regarding their role in PD development, which will help to assess the role and impact of post-transcriptional regulation on disease pathology.

## 1. Introduction

Parkinson’s disease (PD) is a neurodegenerative disease with multiple genetic and environmental factors influencing disease progression. Although the pathophysiology of PD is still not entirely clear, recent studies are going in the direction of uncovering the importance of transcriptional and post-transcriptional regulation in the context of disease pathology. To date, accumulating evidence has shown that genetic variations of microRNA (miRNAs) or miRNA-binding sites could affect miRNA function and contribute to PD risk.

Typical human mRNA consists of a coding sequence immersed between two non-coding parts composed of a cap and a 5′ untranslated region (UTR) (leader sequence) at its start, and a 3′ UTR (trailer sequence) and a poly-A tail at its end. Once thought of as junk mRNA, UTRs are actually very complex structures and serve important gene regulatory roles. While 5′ UTR is necessary for the binding of ribosomes and translation initiation, 3′ UTR has a critical role in translation termination and post-transcriptional modification [1]. The 3′ UTR region is influential in polyadenylation, translation efficiency, localization, and mRNA stability [1].

Because they do not encode for amino acids, 3′ UTRs are rarely included in mutation screenings. However, if we consider their role in translational regulation by providing binding sites for miRNA and RNA binding proteins (RBP), it is clear that variants in 3′ UTRs of PD-associated genes could contribute to neurodegeneration and may regulate the risk for PD [2,3]. Mutations extending 3′ UTR region of α-synuclein protein are implicated as one of the culprits in development of some forms of PD, promoting its accumulation and translation of defective α-synuclein protein [4]. Furthermore, numerous GWAS (Genome-Wide Association Studies) showed strong signals located in the SNCA (Synuclein Alpha) 3′ UTR region [5,6,7,8,9].

Alternative cleavage and polyadenylation are known to produce a great number of SNCA transcripts, which are thought to have a length of 3′ UTR ranging from 290 to 2520 nucleotides [nt] [4,10]. Moreover, some studies associated single nucleotide polymorphisms found in the 3′ UTR SNCA gene with a greater accumulation of long 3′ UTR transcripts resulting in an increase in α-synuclein protein levels [11,12]. Other studies showed a connection between SNCA 3′ UTR length and PD risk [4,10]. The analysis of variants in the 3′ UTR region is complicated by the fact that the length of the region itself is still not completely clear. A recent study identified α-synuclein transcript having a much longer 3′ UTR, with additional 1246 nt [13]. They validated their results in postmortem human brain samples and iPSCs-derived dopaminergic neurons (induced Pluripotent Stem Cells), as well as various human neuronal cell lines [13], questioning what other variants are also part of the 3′ UTR region. Moreover, RNA-seq data uncovered a vast number of previously unannotated 3′ UTR extensions in mice and human [14], while longer transcripts have been shown to be exclusive to brain tissue [13,14]. Thus, this region is interesting because of its role in translation control and genetic variants in 3′ UTR can affect miRNA activity and consequently alter the translation process [15,16]. The aim of this study was to assess differences in 3′ UTR variants of SNCA genes in a cohort of PD patients and control subjects from Croatia.

## 2. Materials and Methods

### 2.1. Patients and Control Subjects

PD patients and healthy control subjects were Caucasians of Croatian origin selected from the University Hospital Center Zagreb. The cohort consisted of 52 PD patients and 23 healthy control subjects (Table 1). All patients were recruited by neurologists from the Department of Neurology at University Hospital Center Zagreb. Each patient underwent a physical and neurological examination. PD was diagnosed according to the UK Parkinson’s Disease Society Brain Bank clinical criteria [17]. None of the control subjects presented with PD symptoms at the age of enrolment. All procedures were approved by the ethical committee of University of Zagreb School of Medicine and University Hospital Center Zagreb and were in accordance with the 1964 Helsinki Declaration and its later amendments or comparable ethical standards. Informed consent was obtained from all individual participants included in the study. 

### 2.2. Sample and Data Collection

Blood samples (3 mL) were collected in EDTA containing tubes from all patients and control subjects. DNA was extracted from whole blood samples following the manufacturer′s specifications using Quick DNA Kit (Zymo Research Corp., Irvine, CA, USA). A NanoDrop 2000 (Thermo Scientific, Wilmington, MA, USA) spectrophotometer and a Qubit 4 (Life Technologies, Carlsbad, CA, USA) fluorometer were used to determine the quality and concentration of genomic DNA samples. Only DNA of OD (optical density coefficient, 260/280) 1.80 ± 20% was used for further experiments.

### 2.3. Next Generation Sequencing

Data for this study were used from data obtained by next generation sequencing of the LYSOGENE gene panel. The next-generation sequencing panel was constructed based on a literature search of the Human Lysosome Gene Database (hLGDB). Relevant genes related to Parkinson’s disease, synucleinopathies, neurodegeneration, autophagic-lysosomal pathways, and lysosomes were included. Based on the data searched, 440 genes were selected. However, for this study, only the 3′ UTR variants of SNCA gene were analyzed. Libraries for next generation sequencing were prepared using the SureSelect QXT Target Enrichment Kit (Agilent technologies, Santa Clara, CA, USA) according to manufacturer′s instructions. Briefly, each DNA sample was diluted, fragmentated and amplified. The amplified samples were re-purified with magnetic particles, and the quality and quantification of the samples was evaluated via a DNA 1000 Kit using Agilent 2100 Bioanalyzer (Agilent technologies, Santa Clara, CA, USA). The fragments were then hybridized and amplified, and the regions of interest were separated using streptavidin magnetic particles. The next step was to add double indexes to name each sample for further recognition, and finally they were diluted to a concentration of 12pM.

The prepared libraries were sequenced on a MiSeq (Illumina, San Diego, CA, USA) next-generation sequencing platform according to the manufacturer′s instructions, generating approximately 5 million of 150-bp paired-end reads for each sample (Q30 ≥ 96%). Considering the technical capabilities of the device and the size of the LYSOGENE panel, we simultaneously sequenced 12 samples. After sequencing, FASTQ files were imported to SureCall software (Agilent technologies, Santa Clara, CA, USA) which was used for further analysis of the sequencing data. The pipeline, after sequencing data submission, included the following steps: quality checks and filter of the reads; alignment on the reference genome, coverage statistics and metrics; variant calling and annotation.

### 2.4. Statistical Analysis

Differences between 3′ UTR allele and genotype frequencies were compared through Chi-squared or Fisher′s exact tests. These were also used to determine whether the observed genotype frequencies differed from those expected under the Hardy–Weinberg equilibrium. All statistical analysis were performed with the Statistica 10 software (StatSoft Inc., Tulsa, OK, USA), and *p* value < 0.05 was considered as statistically significant.

## 3. Results

In our study, we identified four previously reported single nucleotide polymorphisms (SNPs) and one insertion in the 3′ UTR region of the SNCA gene (NM_000345.3): c.*2108A > T (rs1045722), c.*2105G > A (rs3857053), c.*860T > A (rs577490090), c.*893C > T (rs356165) and c.*1309_*1310insTTT (rs777296100), and five variants not reported in the literature: c.*1274_*1277delCTCT (rs35270750), c.*2320A > T (rs529553259), c.*1287G > A (rs377356638), c.*1092delA (rs571454522), and c.*1092_*1093insA (rs750347645) (Table 2).

**Figure 1:**
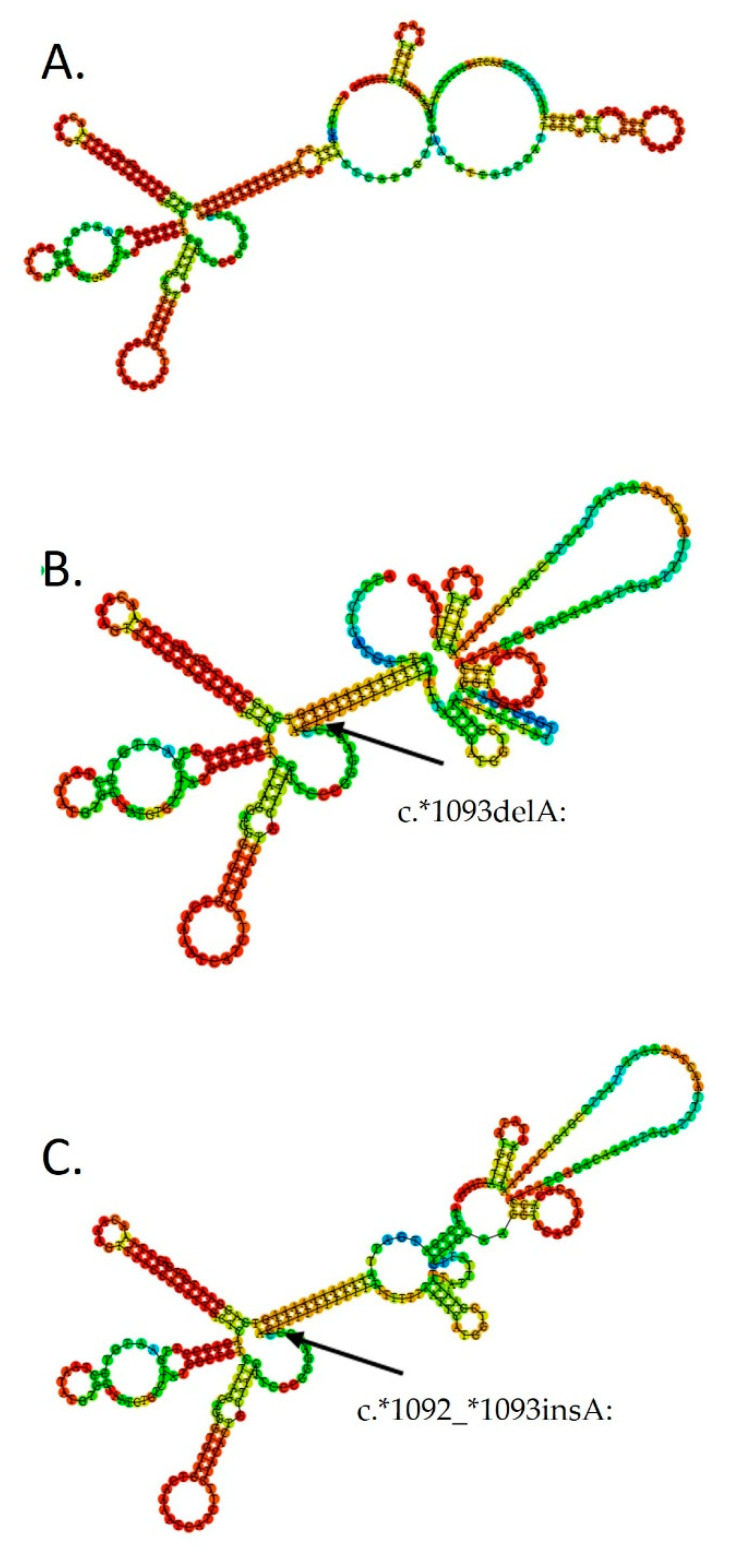
Predicted RNA folding with either the wild tipe (**A**), loss (**B**) or gain (**C**) of rs17016074 for a 300 bp region surrounding the SNP. Structures are obtained from the RNAfold server http://rna.tbi.univie.ac.at/cgi-bin/RNAWebSuite/RNAfold.cgi. (accessed at 26 September 2021).

Our analysis highlighted a higher frequency of rs750347645 in PD patients as compared with healthy control subjects but without statistical significance (*p* = 0.097, Fisher′s exact test). The rs750347645 variant was present in eight PD patient and in no control subjects. However, notably, in novel classification, the rs750347645 variant has merged into rs571454522. rs571454522 was present in 28 PD patients and nine control subjects (*p* = 0.318; Fisher′s exact test). However, if we analyze patients who have rs750347645 (gain) or rs571454522 (loss), there is a significant difference in allele distribution between PD patients and control subjects (36 PD patients and nine control subjects; 0.0211; Fisher′s exact test). To the best of our knowledge, this variant has not been reported until now in the literature. Predicted RNA folding with either a gain or loss in the chr4:89725536-89725550 position is presented in Figure 1.

The rs356165 variant is most commonly described in the context of PD and SNCA polymorphisms in or near the 3′ UTR. Interestingly, our results did not show a difference in variant occurrence in PD vs. control subjects (44/52 vs. 20/23; *p* = 1.000, Fisher′s exact test).

**Figure 2:**
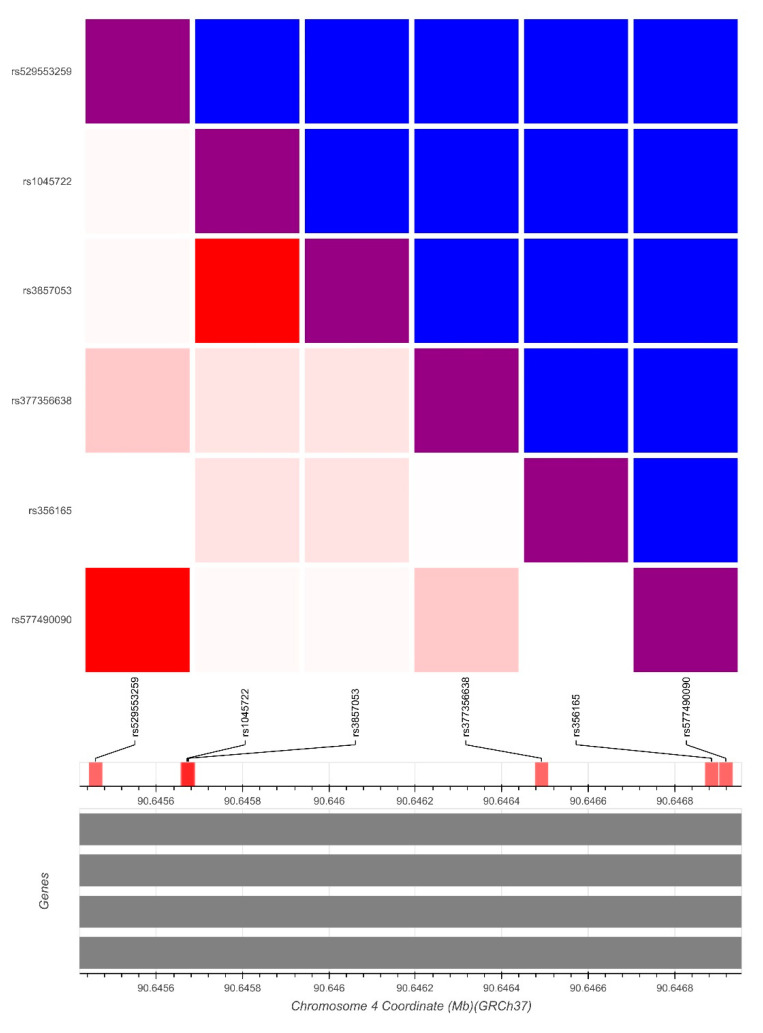
Linkage disequilibrium (LD) structure and physical gene map of the SNCA gene. Blue cells represent D′ correlation; red cells represent R2 correlation. Red cells indicate strong LD; pink cells-intermediate or ambiguous; white cells—evidence for historical recombination. Analysis of linkage disequilibrium of identified variants in the 3′ UTR region of SNCA gene performed via LDmatrix Tool (https://ldlink.nci.nih.gov; accessed at 26 September 2021).

**Figure 3:**
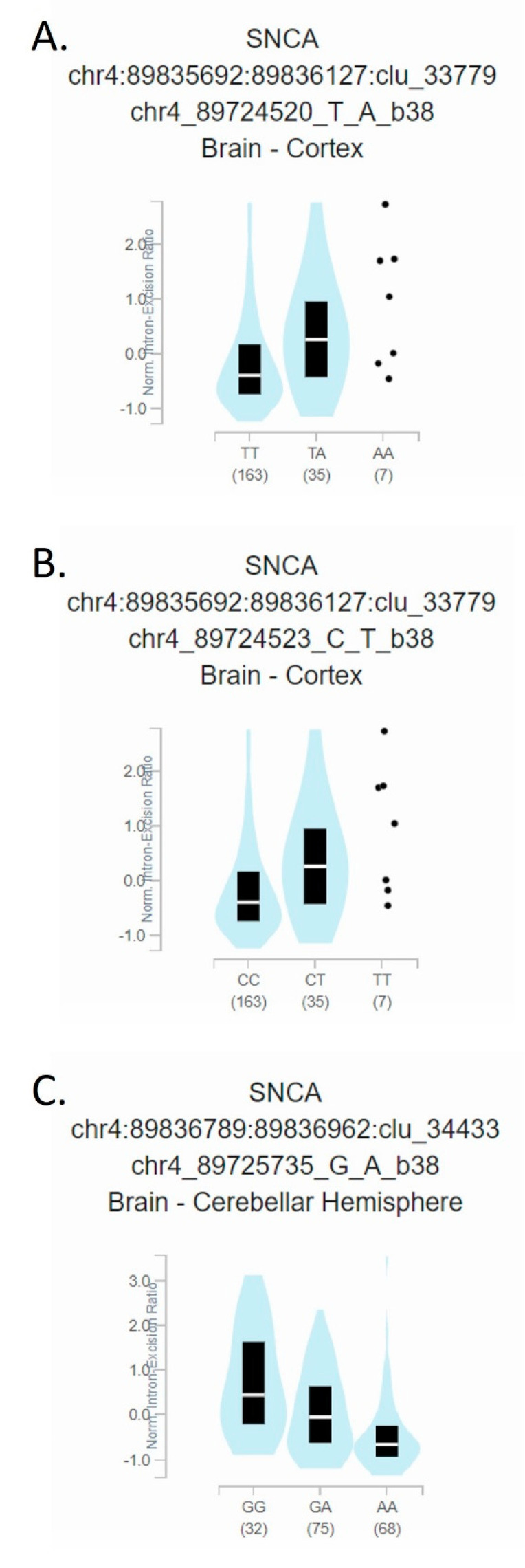
Figure is processed from GTeX (https://www.gtexportal.org; accessed at 26 September 2021 Data Source: GTEx Analysis Release V8 (dbGaP Accession phs000424.v8.p2)), and represent the splicing quantitative trait locus (sQTL) effects of: (**A**). rs1045722 in the 3′ UTR of SNCA and (**B**). rs3857053 in the 3′ UTR of SNCA in cortex and (**C**). rs356165 in the 3′ UTR of SNCA cerebral hemisphere.

In order to approach the question of the mutual influence of individual variants, we analyzed linkage disequilibrium (LD) and showed that the variants rs1045722 and rs3857053 are in complete LD (D′ = 1; R2 = 1), as well as the variants rs577490090 and rs529553259 (D′ = 1; R2 = 1) (Figure 2). Variants rs1045722 and rs3857053 are mutually in complete LD, but also in intermediate LD with rs356165 (D′ = 1; R2 = 0.122). The splicing quantitative trait locus (sQTL) effects of those three variants are presented in Figure 3.

## 4. Discussion

**Figure 4:**
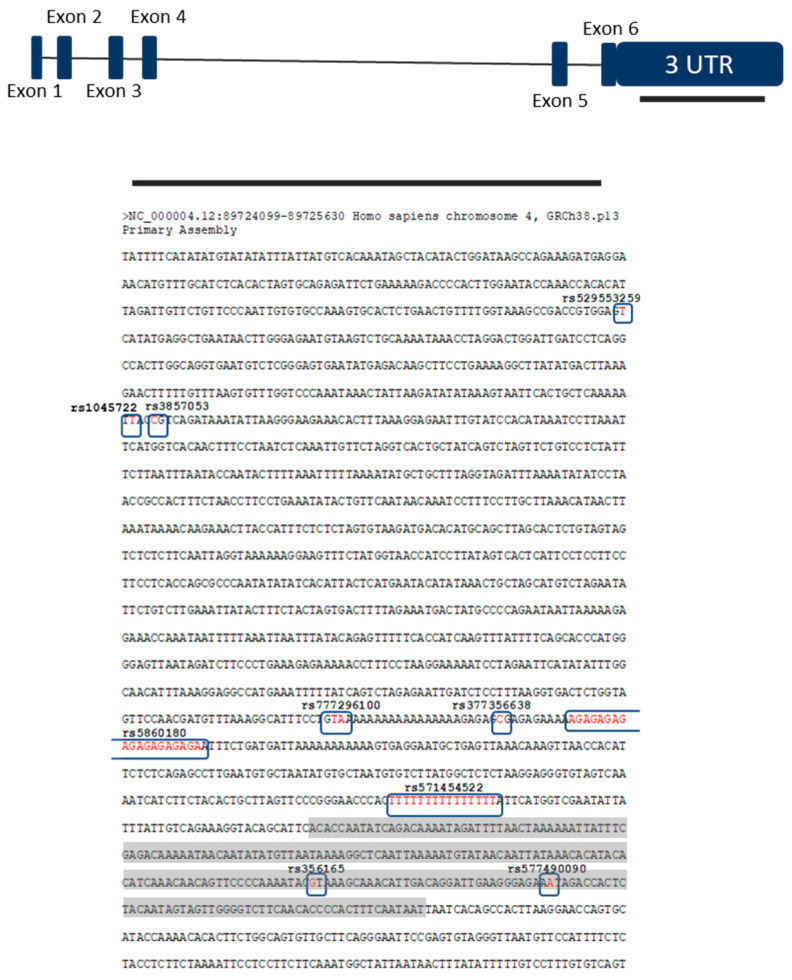
Part of 3′ UTR of the SNCA gene with detected variants from our study. Red nucleotides represent altered alleles. Nucleotides highlighted in grey are the LINE element of 3′ UTR of SNCA gene (226pb) (Sotitiou et al. 2009).

The SNCA gene is a major risk gene for PD and SNCA gene polymorphisms have been associated with the common sporadic form of PD. GWAS reported that variants located in or near the 3′ UTR region of the SNCA gene are significantly associated with PD [5,6,7,8,9]. A study aimed at better understanding the role of epigenetic influences showed higher expression of SNCA-targeting miRNAs in gyri cinguli of patients and controls [18]. On the other hand, Kabaria and colleagues [19] have found that rs10024743in the SNCA 3′ UTR lowers the miR-34b-mediated repression of the protein and suggested that down-regulation of miR-34b and miR-34c in the brain can increase α-synuclein expression, possibly contributing to PD pathogenesis. SNCA SNP allele frequencies vary widely among populations (allele frequencies of detected variants from our study population are presented in Table 2). We focused on the 3′ UTR region of SNCA as it is thought to perform post-transcriptional regulation of SNCA expression. We observed a higher prevalence of some variants in 3′ UTR region among PD patients compared with control subjects and compared our results with other studies. A recent association study performed on data from the largest GWAS of miRNA-related genetic variants showed that three PD-associated SNPs (rs356165, rs3857053, and rs1045722) in the SNCA 3′ UTR overlap with poorly conserved miRNA-binding sites [20]. Furthermore, Toffoli et al. found that the same three SNCA-3′ UTR SNPs are more frequent in PD patients than in RBD (Rapid-Eye-Movement Sleep Behaviour Disorder) patients (*p* = 0.014, 0.008, and 0.008, respectively) [21]. On the other hand, our results did not show a different distribution of those SNPs between PD patients and healthy control subjects in our population (Table 2). In our study rs356165 was present in 44 PD patients and 20 control subjects (Chi Squared = 0.05; *p* = 0.94), while rs3857053 and rs1045722 were present together in same 4 PD patients and 3 control subject (Fisher′s exact test, *p* = 0.6687). SNP rs356165 is located in the 3′ UTR of SNCA and is one of the top associated SNPs with PD in the related locus [20]. Ghanbari et al. found that the rs356165 mutant allele is predicted to disrupt a binding site of miR-6508, presumably resulting in an elevated level of SNCA expression [20]. Cardo et al. showed a significant association of rs356165 with PD risk in a Spanish cohort (*p* = 0.0001; odd ratio = 1.37, 95%CI = 1.19–1.58) [12]. They also found a significant correlation with early age at onset of PD, highlighting rs356165 as an important risk factor for PD. Emelyanov confirmed that G alleles of rs356165 are associated with increased risk of PD development [22]. Both the level of mRNA SNCA gene and that of α-synuclein protein were increased in rs356165 carriers compared to carriers with AA genotype in control group (*p* = 0.046 and *p* = 0.039, respectively) [22]. As can be seen from the above, numerous studies showed a strong association of the rs356165 variant with sporadic form of PD, although the results are not entirely consistent. A case control study of 330 PD patients and 300 control subjects did not reveal a statistically significant difference in genotype frequency between cases and controls (*p* = 0.863), indicating that there is no association of SNP rs356165 with PD in the Chinese population [23]. Figure 4 shows that rs356165 is present in the LINE (long interspersed nuclear element) of 3′ UTR of human SNCA and outside the initial 574nt, so it could be concluded that it is involved in only 5% of SNCA mRNA [11]. Rs356165 may therefore have a direct effect on SNCA expression but affecting only a small percentage of the SNCA mRNA in which it is present. However, it should be emphasized that extended transcripts with longer 3′ UTR have a higher prevalence in brain tissue and thus variants in this region may have a greater impact in central nervous system. Nevertheless, the association of rs356165 and sporadic PD could be explained by other variants that are in linkage disequilibrium with rs356165. Our result showed intermediate LD of variants rs1045722 and rs3857053 with rs356165 (R2 = 0.122; Figure 2 and Figure 3).

Rudakuou and colleagues detected two SNCA 3′ UTR variants, rs1045722 and rs3857053 in the 3′ UTR of SNCA [9]. Analysis showed that they were in between in full LD, and in strong LD with rs356219, a known 3′ GWAS hit in the SNCA locus [9]. This is in accordance with our results (Figure 2). These two variants are located in an enhancer region and are associated with altered splicing of SNCA in the cortex [9]. Furthermore, Tagliafierro found that the short-structural variant rs777296100-polyT was moderately associated with dementia with Lewy bodies but not with PD which is in accordance with our results [24]. We found a polyT extension (c.*1309_*1310insTTT) (rs777296100) in 31 PD patients and 13 control subjects. Shen and al showed that MiR-30b may bind to the 3′ UTR side of SNCA and inhibited its expression [25]. On the other hand, Schmitt and al found two rare variants, rs145304567 and c.*1004_1008delTTTTT [26]. The variant rs145304567 affects the binding site of miRNA -433. However, they found a similar allele distribution in PD patients and controls [26]. Kabaria identified a polymorphic variation (rs10024743) in the 3′ UTR of human SNCA gene, suggesting that the rs10024743 SNP could be a cause of resistance to miR-34b-mediated repression of SNCA expression, resulting in an increase of α-synuclein expression [19].

An interesting result from our study was the distribution of a novel variant rs750347645. Our results showed a higher frequency of rs750347645 in PD patients as compared with healthy control subjects (*p* = 0.097), but without statistical significance. The rs750347645 variant was present in eight PD patients and in no control subjects. In novel classification in the dbSNP database, the rs750347645 variant has merged with rs571454522. If we analyze patients who have rs750347645 (gain) together with patients with rs571454522 (loss) there is a significant difference in allele distribution between PD patients and control subjects (36 PD patients and nine control subjects; 0.0211; Fisher′s exact test). To the best of our knowledge, this variant has not been reported in the literature until now and further research is needed to assess its significance in PD pathology.

Recent evidence suggests an association between PD and SNCA isoforms with 3′ UTR of different lengths. A recent study showed a higher level of SNCA transcripts with different 3′ UTR lengths in the cerebral cortex samples of postmortem patients with PD [4]. On the other hand, another study associated significantly lower levels of SNCA mRNA with prolonged 3′ UTR in motor forms of PD [27]. Depending on the choice of polyadenylation site, human SNCA mRNA is expressed in five isoforms of different 3′ UTR lengths (ranging from 290 nt to 2500 nt; 290 nt, 480 nt, 575 nt, 1074 nt, 2530 nt) [4,10]. Among different isoforms, the one carrying 3′ UTR of 575 bases was found to be the most abundant in SH-SY5Y and HeLa cells (∼50% of total SNCA mRNA) [10]. It was observed that the short isoform 3′ UTR is more active than the longer isoforms and significantly contributes to protein synthesis in vivo [10]. Sotiriou and colleagues showed that the first 574 nucleotides of the 3′ UTR of human SNCA are highly conserved and included in 95% of its mRNAs [11]. Still, as mentioned above, research showed that transcripts with long 3′ UTR have a higher prevalence in brain tissue and thus may have a greater impact on the neural system or a neuron-specific function [14]. Research has also identified that the rs17016074 variant can increase reporter gene expression in neuroblastoma SHSY5Y cells [11]. We did not find the rs17016074 variant in our population, and interestingly all the variants described in our results are located outside the first 574 nt (Figure 4).

There are several limitations to our study. One limitation is sample size, and data from additional participants may yield new insights. However, the results we obtained are the first results of this type in our Croatian population, and the sample size was still sufficient for the identification of a significantly higher occurrence of the rs571454522 variant in the PD population. Another limitation of this study is the lack of mechanistic validation of our results. To try to overcome this, we conducted additional analyses on prediction software, and we tested the impact on splicing and analyzed linkage disequilibrium. Furthermore, our research was conducted exclusively from a genomic point of view, but we have put our results in the context of other research focusing on their mechanistic findings and discussed the results from a functional aspect.

## 5. Conclusions

In conclusion, further studies will be needed to gain a more profound insight into the role of 3′ UTR variants in PD development, which will help to assess the role and impact of post-transcriptional regulation on disease pathology.

## Figures and Tables

**Table 1:** Demographic description of the study subjects.

	PD	M	F	Control	M	F
Number of subjects	53	29 (54%)	24 (46%)	20	10 (50%)	10 (50%)
Mean age (SD)	66	63	70	63	64	63
Median age ± SD	66.33 ± 8.63	63.68 ± 8.07	69.86 ± 8.23	64.56 ± 9.19	62.43 ± 9.18	64.5 ± 8.37

SSD—statistic deviation, M—male, F—female.

**Table 2:** Identified variants in the 3′ UTR region of SNCA gene.

ID	PD	CTRL	*p* Value *	HGVS (NM_000345.3)	Position GRCh38.p13	Alleles		Type	Allele Frequency (1000 G)
rs1045722	4	3	0.6687	c.*2108A > T	chr4:89724520	T > A/T > G	[15]	SNP	0.38
rs3857053	4	3	0.6687	c.*2105G > A	chr4:89724523	C > T	[15]	SNP	0.377
rs777296100	31	13	0.8052	c.*1309_*1310insTTT	chr4:89725318-89725319	insTA/insTAA/	[16]	Insertion	
rs35270750 (rs5860180)	37	16	1.000	c.*1274_*1277delCTCT	chr4:89725351-89725369	delGAGA/	NR	Deletion	0.331
rs356165	44	20	1.000	c.*893C > T	chr4:89725735	G > A	[15]	SNP	0.484
rs529553259	1	0		c.*2320A > T	chr4:89724308	T > A	NR	SNP	0.001
rs377356638	1	0		c.*1287G > A	chr4:89725341	C > T	NR/LB	SNP	0.046
rs577490090	1	0		c.*860T > A	chr4:89725768	A > T	NR/VUS	SNP	0.001
**rs571454522**	**36**	**9**	**0.0211**	c.*1092delA/insA	**chr4:89725536-89725550**		**NR**	**Deletion/insertion**	
rs571454522 (loss)	28	9	0.318	c.*1092delA	chr4:89725536-89725550	delT	NR	Deletion	
rs750347645 (rs571454522) (gain)	8	0	0.0974	c.*1092_*1093insA	chr4:89725536-89725550		NR	Insertion	

* Fisher′s exact test. Rs number in brackets indicates the new rs number for the same variant. CTRL control. B benign. LB likely benign. VUS variant of uncertain significance. NR not reported. Bold are marked as sum of the two following rows.

## Data Availability

The data presented in this study are available on request from the corresponding author.
